# Male Breast Cancer and Hyperestrogenemia: A Thirteen-Year Review

**DOI:** 10.14740/wjon803w

**Published:** 2014-05-06

**Authors:** Khurram Bilal Tariq, Farah Al-Saffar, Saif Ibrahim, Dat Pham, Arezo Farhangi, Fauzia Rana, Robert Zaiden

**Affiliations:** a Department of Internal Medicine, University of Florida, College of Medicine, 653 West 8th Street, Box L 18, Jacksonville, FL 32209, USA; b Division of Hematology and Medical Oncology, University of Florida, Jacksonville, FL, USA

**Keywords:** Male breast cancer, High circulating estrogen

## Abstract

**Background:**

Male breast cancer (MBC) is a very rare malignancy and accounts for 0.1% of all male cancers. MBC has not been studied as extensively as its female counterpart. Certain clinical and pathological risk factors like smoking history, age at onset, family history of cancer, obesity, ethnicity, estrogen/progesterone receptor status and BRCA gene mutation status have all been studied well in the female breast cancer (FBC) patients and the clinical trial evidence from these studies is then extrapolated to treat and manage patients with MBC. One such area of interest is high levels of estrogen and its relationship with MBC. In our retrospect research study we aim to find an association between MBC and high levels of circulating estrogen at the time of diagnosis.

**Methods:**

A 13-year retrospective review of the male breast cases at University of Florida College of Medicine’s Tumor Registry was conducted. Data regarding certain clinic-pathological risk factors and MBC were collected and reviewed. Main surrogate indicators for elevated estrogen were examined, namely, low HDL (< 40 mg/dL), low albumin (< 4 g/dL) and high BMI (> 25). Presence of any one of these surrogates was seen as an indirect marker for high estrogen level. For cancer staging, the American Joint Committee on Cancer (AJCC) staging system was used. Stages 0-2 were grouped together as they are less extensive compared to stages 3-4 (also grouped together) which represent extensive disease. Univariate analysis was conducted using STATA 13 to do Fischer’s exact test as cross-tables showed cell counts of five or less. The main comparison was that between extensive MBC (stages 3-4) and non-extensive breast cancer (stages 0-2).

**Results:**

Between January 2000 and November 2013, we found a total of 2,129 cases of breast cancer patients at our institute. Out of these 2,113 (99.24%) were female and 16 (0.75%) were men. Four MBC patients were excluded because their complete charts could not be found in the medical records department. Six (50%) patients had one indicator, four (33%) patients had two indicators and one (8.3%) patient had all three. Eleven (91.6%) patients had precursors suggestive of hyperestrogenemia. Only one (8.33%) patient did not have any surrogate marker indicator of high estrogen levels. Two (16%) were black and 10 (83.33%) were white. Mean age was 61.75. Five (41%) had a first degree relative with a malignancy. Laterality was nine (75%) in the left breast, three (35%) in right breast. Eight (66.6%) found a mass on physical exam. Five (41.6%) had a positive smoking history. One patient had no data in the chart. Remaining all 11 (91.6%) had non-TNBC. One patient did not have complete documentation. Five (41.6%) had mastectomy, six (50%) received RT, four (33.3%) received chemotherapy and another four received hormone therapy. In terms of stage, four (33.3%) had stage 4, two (16.6%) stage 3B, two (16.6%) stage 2B, two (16.6%) stage 2A, one (8.33%) had stage 1C and one had stage 0. HDL data were available in seven (58.3%) with mean of 37, albumin in 10 (83.3%) with mean of 3.61, BMI in 11 (91.66%) patients with a mean of 33.30. Within subgroups, two patients were black and 10 white. Both black patients had LE disease (stage 0-2). Of the white patients, four (40%) had limited disease while six (60%) had extensive breast cancer. Family history assumed a similar distribution as three (60%) of patients with negative family history for cancer had limited disease and two (40%) had extensive one, same numbers applied for family-history-positive population. Three (60%) of patients with limited disease smoker and two (40%) did not. As for laterality, a total of nine patients had left-sided breast cancer, of whom five had had limited disease and four fell into the extensive disease category. The hormonal status for most patients were HER/NEU negative (seven out of 10 patients, two patients did not have this information on file), ER positive (11 out of 12) and PR positive (8 out of 12). Estrogen status: Low HDL was seen in three out of seven patients, low albumin in four out of 10 and obese BMI in nine out of 11. Finally, 11 out of 12 patients had at least one indicator of high estrogen. No significant change in prevalence of these markers was seen when comparing patients with limited and extensive disease.

**Conclusion:**

None of the aforementioned variables assumed statistical significance between the two subgroups. Results, however, show that as a whole, 11 out of the 12 patients had at least one indicator of high estrogen. Our results point in the direction that elevated estrogen is probably associated with MBC. Further meta-analysis of similar studies can be helpful to explain the dynamics of this association. Our statistical analysis was limited due to the small sample size, which is due to the extreme rarity of the disease.

## Introduction

Breast cancer in males is a fairly rare malignancy and accounts for 0.1% of all malignancies in men and less than 1% of all breast cancers [1-4]. It also carries a significantly higher mortality rate when compared with the breast cancer in female patients [1]. There are several factors which might contribute to this special finding. Unlike the epidemiological variations in trends seen over the past few decades in female breast cancer (FBC), the incidence of breast cancer in males has not increased and even large oncology centers tend to have small number of male breast cancer (MBC) patients. As a society, it often comes as a surprise to many that men can also have breast cancer [5]. It is therefore not surprising that there exists a lack of research on MBC when compared to its more common and widely studied counterpart, the FBC. As a result, most of our management of MBC is actually an extrapolation of the research carried out on the FBC. While this offers an intuitive and practical means of combating a malignancy that is comparatively rare in men, this lack of research fails to account for the discovery and analysis of any variations in MBC which may require different treatment modalities [1, 6, 7]. While the role of estrogen in FBC is well established, there exists a paucity of research on the association between high circulating estrogen and MBC. Our retrospective study aims to specifically look at this paradigm.

## Materials and Methods

The present study involves a 13-year retrospective review of the male breast cases at University of Florida College of Medicine in Jacksonville’s Tumor Registry was conducted. Data regarding certain clinic-pathological risk factors and MBC were collected and reviewed. Main surrogate indicators for elevated estrogen were examined, namely, high HDL (> 40 mg/dL), low albumin (< 3.5 g/dL) and high BMI (> 25). Presence of any one of these surrogates was seen as an indirect marker for high estrogen level. Univariate analysis using chi-square test and Fisher exact test were used to compare possible predictors. The predictors analyzed included the general characteristics (age, smoking, family history of cancer, tumor receptor status) as well as high estrogen indicators mentioned above. None of these predictors showed a statistical significance, which could be attributed to the small sample size secondary to the rarity of this disease. These patients were divided into two subgroups with stages 0-2 in one group and stages 3-4 in the other group. These groups will be referred to as less extensive (LE) and more extensive (ME) MBCs, respectively.

## Results

**Figure 1:**
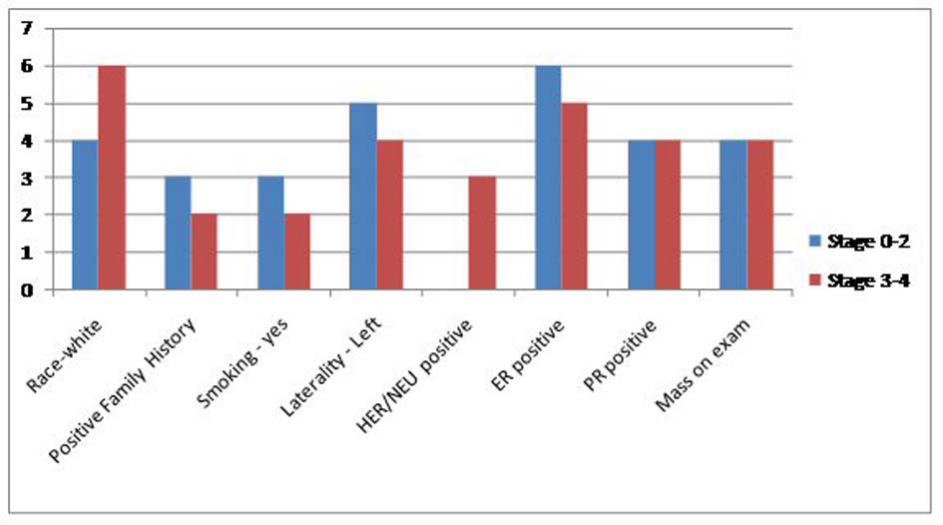
Comparison of the general characteristics of the study population between extensive and non-extensive stages of MBC.

**Figure 2:**
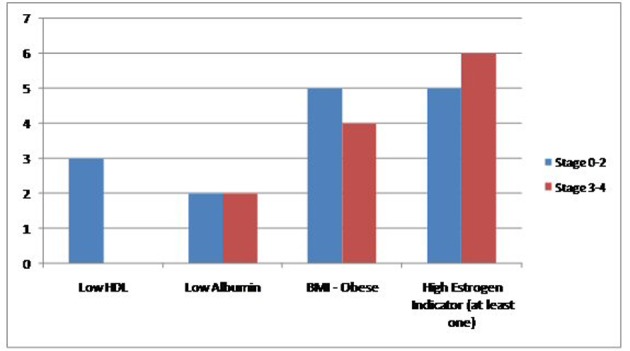
Comparison of the prevalence of high estrogen indicators in the study population between extensive and non-extensive stages of MBC.

Between January 2000 and November 2013, we found a total of 2,129 cases of breast cancer patients at our institute. Out of these 2,113 (99.24%) were female and 16 (0.75%) were men. Four MBC patients were excluded because their complete charts could not be found in the medical records department. Six patients (50%) had one indicator, four (33%) patients had two indicators and one (8.3%) patient had all three. Eleven (91.6%) patients had precursors suggestive of hyperestrogenemia. Only one (8.33%) patient did not have any surrogate marker indicator of high estrogen levels. Two (16%) were black and 10 (83.33%) were white. Mean age was 61.75. Five (41%) had a first degree relative with a malignancy. Laterality was nine (75%) in the left breast, three (35%) in right breast. Eight (66.6%) found a mass on physical exam. Five (41.6%) had a positive smoking history. One patient had no data in the chart. Remaining all 11 (91.6%) had non-TNBC. One patient did not have complete documentation. Five (41.6%) had mastectomy, six (50%) received RT, four (33.3%) received chemotherapy and another four received hormone therapy. In terms of stage, four (33.3%) had stage 4, two (16.6%) stage 3B, two (16.6%) stage 2B, two (16.6%) stage 2A, one (8.33%) had stage 1C and one had stage 0. HDL data were available in seven (58.3%) with mean of 37, albumin in 10 (83.3%) with mean of 3.61, BMI in 11 (91.66%) patients with a mean of 33.30. Within subgroups, two patients were black and 10 white. Both black patients had LE disease (stage 0-2). Of the white patients, four (40%) had limited disease while six (60%) had extensive breast cancer. Family history assumed a similar distribution as three (60%) of patients with negative family history for cancer had limited disease and two (40%) had extensive one, same numbers applied for family-history-positive population. Three (60%) of patients with limited disease smoker and two (40%) did not. As for laterality, a total of nine patients had left-sided breast cancer, of whom five had had limited disease and four fell into the extensive disease category. The hormonal status for most patients were HER/NEU negative (seven out of 10 patients, two patients did not have this information on file), ER positive (11 out of 12) and PR positive (eight out of 12). Estrogen status: Low HDL was seen in three out of seven patients, low albumin in four out of 10 and obese BMI in nine out of 11 patients. Finally, 11 (91.6%) out of 12 patients had at least one indicator of high estrogen. No significant change in prevalence of these markers was seen when comparing patients with limited and extensive disease. Further details are presented in Figures 1, 2, and Tables 1, 2.

**Table 1:** Comparison of the General Characteristics of the Study Population Between Extensive and Non-Extensive Stages of Male Breast Cancer, Percentages Are Included in Parenthesis

	Stage 0-2 (%)	Stage 3-4 (%)	P value
Race			0.455
Black	2 (100)	0 (0)	
White	4 (40)	6 (60)	
Family history			1
Negative	3 (60)	2 (40)	
Positive	3 (60)	2 (40)	
Smoking			1
No	3 (50)	3 (50)	
Yes	3 (60)	2 (40)	
Laterality			1
Right	1 (33.3)	2 (66.6)	
Left	5 (55.5)	4 (44.4)	
HER/NEU			0.167
Negative	5 (71.4)	2 (28.5)	
Positive	0 (0)	3 (100)	
ER			1
Negative	0 (0)	1 (100)	
Positive	6 (54.5)	5 (45.4)	
PR			1
Negative	2 (50)	2 (50)	
Positive	4 (50)	4 (50)	
Mass on PE			1
No	1 (50)	1 (50)	
Yes	4 (50)	4 (50)	
Low HDL			0.143
No	1 (25)	3 (75)	
Yes	3 (100)	0 (0)	
Low albumin			1
No	3 (50)	3 (50)	
Yes	2 (50)	2 (50)	
BMI			1
Normal	1 (50)	1 (50)	
Obese	5 (55.5)	4 (44.4)	
High estrogen score			1
None	0 (0)	1 (100)	
At least one	5 (45.5)	6 (54.5)	

**Table 2:** Summary of the Treatment Received for Male Breast Cancer, Summarized by Cancer Stage

	Stage 0-2 (Less extensive group)	Stage 3-4 (More extensive group)
Surgery		
No	2	4
Yes	3	2
Beam radiation		
No	2	2
Yes	3	3
Chemotherapy		
No	2	3
Yes	3	1
Hormonal therapy		
No	2	2
Yes	4	3

## Discussion

Dividing the patient population into two subgroups based on the severity of their stage (LE vs. ME groups) our study failed to yield any statistically significant outcomes. These details are elaborated in the results (Fig. 1, 2) (Table 1, 2). Therefore, the discussion below will be based on the descriptive analysis of the total MBC populations as a whole and comparisons will be made with research done elsewhere to better ascertain our findings. Several factors contribute to the higher mortality rates associated with MBC compared to the female subtype. Incidence of MBC has stayed stable over several decades; however, unlike the bimodal peaks seen in FBC, the unimodal incidence of MBC increases with advanced age. Unlike the hormonal fluctuations after menopause seen in women, there are no hormonal fluctuations in men and this may account for their unimodal peak [8]. The mean age at diagnosis in our retrospective study was 61.5, which goes well with other research studies describing the late presentation of MBC [1, 9]. MBC patients also present at a higher stage [4, 10]. In present study a third of our patients presented at stage 4. Most of these patients only sought medical attention after discovering a grossly palpable mass on physical exam. Lack of awareness of the disease and the absence of screening programs for MBC may explain some of these findings. In our patient population these two factors played a crucial role for their delay in seeking attention. In our study only one patient (8.66%) had carcinoma in situ which is comparable to the published literature where the prevalence of MBC in situ is also very small and is reported to be around 11% [9].

Seventy-five percent of our patient population had a mass palpable on physical exam and in 75 of these patients the breast cancer was localized to the left breast. The asymmetrical distribution of certain malignancies including melanomas and mammary carcinomas is an area under research. While in recent years we have made tremendous progress in our knowledge of the molecular control of embryonic symmetry, not much is known about the distributional disparity in disease predominantly seen on the left or right sided of the body [11]. In terms of the breast cancer, on average there is a felt to be a small difference in size with the left breast being slightly larger compared to the right; however, in a retrospective review of 250,000 breast cancer patients Weiss et al were able to disprove such an association [12]. Another possible explanation is the detrimental effect of certain radiofrequency wavelength used in western FM/TV bandwidths. Research points to the increased incidence of breast cancers and melanomas in areas with multiple FM/TV transmitters with higher frequency bandwidths. Sweden, for example, like most of the “developed” countries uses a higher frequency band width of 87 - 108 MHz and has a higher incidence of left laterality then compared to Japan, which is also a “developed” country but where a lower frequency band width of around 76 - 93 MHz is used [13].

In our retrospective review, 41% of the patients had a positive family history of any type of cancer. The association between positive family history of breast and ovarian cancer is reported in about 15-20% in the published research. BRCA2 gene mutation is the most commonly identified genetic mutation in men and it is also shown to increase the lifetime risk of breast cancer in men by about 5-10% compared to the minimal risk of 0.1% seen in the general population [9].The higher proportion seen in our patients may be due to the inclusion of all cancer types compared to only the breast and ovarian cancers seen in the other studies.

In terms of demographics, 83.3% of our patient population comprised of white men whereas only 16% were black. This is in contrast with the published research showing a higher prevalence of MBC in black men of all ages. Interestingly, black women have a lower incidence of breast cancer compared to their white counterparts [9]. Social disparities, including limited access to healthcare and lack of awareness about this rare malignancy might explain the trend seen at our institution.

Smoking history is another important risk factor for consideration in MBC patients. It has a protective role through its anti-estorgenic effect and at the same time the smoke in cigarettes is considered carcinogenic. Several studies have shown mixed results and based on its dual effect on the pathophysiology of breast cancer, it is not difficult to foresee why [14]. In our patient population 41.5% of the MBC patients had a long term history of current or past cigarette smoking.

In terms of its molecular subtypes, the triple negative breast cancer (TNBC) is more common in the black women compared to the non-TNBC subtype which is more common in white females. TNBC also carries the worse prognosis of the two [14]. Most breast cancers in men are the non-TNBC subtype. As many as 85% of the MBC patients are estrogen receptor positive whereas 70% are progesterone receptor positive [4, 15]. In our retrospective review we found that about 91.6% of our patient population had the non-TNBC. This goes well with the published research discussed above.

Most significant part of our thirteen-year retrospective review is the findings pertaining to hyperestrogenemia. The three surrogates we used in the present study were elevated BMI, low HDL and low albumin. A BMI of greater than 25 was used as a marker for obesity. In FBC patients with higher BMIs there are higher levels of circulating estrogen resulting from higher conversions in the adipose tissue. This may lead to anovulatory states and is therefore protective against FBC. After menopause, however, obese women have a higher incidence of breast cancer from higher levels of circulating estrogen [16-18]. In the current study 91.6% of our patient population was considered obese, with a mean BMI of 33.3.

A second surrogate for high circulating estrogen in our study was low HDL level which we defined as a value of less than 40. Studies have shown an increase risk of breast cancer with low HDL in both pre- and postmenopausal women [19]. The mechanism of action of HDL on breast cancer is unclear and some researchers have hypothesized that the low HDL may rather be the result of accompanying metabolic syndrome [20]. Others have shown that low levels of HDL are associated with higher levels of circulating estrogen which has an established role in the carcinogenesis of breast cancer [21]. To add to the complexity, there is even evidence that HDL is independently associated with risk of breast cancer especially in postmenopausal women [20]. In our study HDL data were available in seven (58.3%) patients with a low mean value of 37.

The third surrogate for hyperestrogenemia in our study was an albumin level less than 4.0. In men estrogen is derived from the peripheral conversion in adipose tissue and from testicular sources. This estrogen is then inactivated in the live. It has long been established that liver dysfunction, such as cirrhosis, can lead to higher levels of circulating estrogen which can then cause gynecomastia and hypogonadism. Since albumin is produced in the liver, a decreased in albumin levels in an adult male with no other identifiable basis for this finding is used as a surrogate for liver dysfunction [22]. The mean value of albumin in our patient population was 3.7 which is lower than defined parameters for this study.

Addressing the three surrogates together, we found that in our patient population six patients (50%) showed presence of one of the three surrogates, four (33%) patients had two surrogate markers and one (8.3%) patient had all three. Most importantly, 11 patients which comprised about 91.6% of our patient population, had at least one surrogate marker suggestive of hyperestrogenemia. These are very important findings and suggest the need for bigger, multicenter studies looking into the association between MBC and elevated serum estrogen levels.

Univariate analysis using chi-square test and Fisher exact test were used to compare possible predictors which included the general characteristics (age, smoking, family history of cancer, tumor receptor status) as well as high estrogen indicators mentioned above. None of these predictors showed any statistical significance, which could be attributed to the small sample size owing to the rarity of this disease.

Furthermore our study points towards some key quality improvement objectives at our hospital. Out of the 16 patients who were found to have MBC, medical records of four could not be located. Steps such as electronic conversion of these medical records will help with easier and quick access of medical records in the future.

### Conclusions

MBC is a rare malignancy which has contributed to the paucity of research associated with this malignancy. Results from research studies on FBC are usually inferred to the MBC patient management and treatment. This approach is not helpful in discovering variations that may exist in men with breast cancer for which different treatment modalities may be necessary. One such area of interest is the relationship between hyperestrogenism in men with the onset of MBC. Our results point in the direction that elevated estrogen is probably associated with MBC. Further meta-analysis of similar studies might be helpful to explain the dynamics of this association. Our statistical analysis was limited due to the small sample size, which is due to the extreme rarity of the disease. Our results also emphasize the need for adequate labeling and conversion of paper-based medical record to the electronic form for quick retrieval and ease of analysis in original research studies like ours.
